# A conceptual framework for core stabilization training based on integrated ultrasonographic and functional evidence

**DOI:** 10.3389/fspor.2026.1913798

**Published:** 2026-09-17

**Authors:** Paweł Niewiadomy, Katarzyna Szuścik-Niewiadomy, Michał Kochan, Michał Kuszewski

**Affiliations:** 1 Department of Balneoclimatology and Biological Regeneration, School of Health Sciences in Katowice, Medical University of Silesia in Katowice, Katowice, Poland; 2 Department of Adapted Physical Activity and Sport, School of Health Sciences in Katowice, Medical University of Silesia in Katowice, Katowice, Poland; 3 Institute of Physiotherapy and Health Sciences, The Jerzy Kukuczka Academy of Physical Education in Katowice, Katowice, Poland

**Keywords:** abdominal muscles, core stability, motor control, trunk stabilization, ultrasonography

## Abstract

This manuscript presents a conceptual and integrative framework for understanding method-specific structural and functional adaptations resulting from different core stabilization training approaches in healthy adults. The synthesis is based on harmonized outcome measures derived from three prospective 4-week intervention studies conducted within a single research program. Identical ultrasonographic measurement protocols and standardized trunk mobility assessments were applied across all interventions, enabling a structured examination of adaptation patterns across training models. No pooled re-analysis of individual-level data or direct inferential comparison between training models was performed, and statistical interpretations correspond to those reported in the original trials. By combining ultrasonographic assessment of lateral abdominal wall musculature with functional mobility outcomes, the findings suggest distinct adaptation profiles associated with local, global, and rotational stabilization strategies rather than the universal superiority of any single approach. The proposed conceptual framework organizes structural and functional adaptation patterns within a coherent, mechanism-oriented structure and provides a basis for future hypothesis-driven research in rehabilitation and performance contexts. However, as the evidence is derived from healthy populations within a single research program, direct applicability to clinical populations requires further independent validation.

## 1 Introduction

This manuscript presents a conceptual, integrative framework for core stabilization training derived from a structured synthesis of previously published intervention studies conducted within a single research programme. Rather than introducing new experimental data, the aim is to organize harmonized structural and functional outcomes into a conceptual framework intended to generate hypotheses, support future research on core stabilization training, and inform physiotherapists' clinical reasoning.

The core region is commonly understood as an integrated anatomical and functional system comprising the lumbar spine, pelvis, hip complex, abdominal wall, paraspinal muscles, diaphragm, pelvic floor, and associated fascial structures. Rather than representing a single muscle group, the core functions as a coordinated neuro-muscular system responsible for maintaining lumbopelvic stability, transferring forces between the upper and lower limbs, and supporting controlled movement during both low-load daily activities and demanding physical tasks (1–3). Within this system, the transversus abdominis (TrA), internal oblique (IO), external oblique (EO), multifidus, diaphragm, and pelvic floor contribute to trunk stiffness regulation, intra-abdominal pressure modulation, segmental control, and anticipatory postural adjustments (4–6).

Core stability is a key component of optimal human movement and plays a fundamental role in injury prevention and rehabilitation. It refers to the ability to maintain stability of the lumbopelvic–hip complex during daily activities as well as during demanding physical tasks. Impairments in core stability are commonly associated with low back pain (LBP) and are frequently accompanied by reduced mobility and postural dysfunctions (1–3). Impairments in core function have also been associated with altered trunk motor control, impaired movement coordination, and delayed anticipatory activation of deep abdominal muscles. However, the relationship between structural muscle adaptations, neuromuscular function, mobility outcomes, and clinical symptoms remains debated (6, 7).

The transversus abdominis (TrA) plays a crucial role in trunk stabilization due to its anticipatory activation during motor tasks (4–6). Delayed activation or reduced functional capacity of the TrA—particularly in individuals with LBP—may contribute to segmental instability and secondary muscular compensations (7, 8).

To address these impairments, several core stabilization training models have been developed, including local (LT), global (GT), and rotational training (TG), each based on distinct principles of muscle activation. Local training focuses on isolated activation of deep trunk muscles, primarily the TrA and multifidus, using anticipatory feedforward strategies and is commonly applied in patients with chronic low back pain (9, 10). Global training emphasizes activation of superficial trunk muscles, such as the rectus abdominis, external oblique (EO), and erector spinae, supporting dynamic stabilization through multi-muscle movement patterns (11, 12). Rotational training involves transverse-plane movements engaging the oblique abdominal and paraspinal muscles, thereby enhancing trunk mobility and rotational stability (13–15). Various additional approaches to core-oriented training, including hypopressive and Pilates-based interventions, have also been described in the literature (16, 17). However, the present framework focuses specifically on local, global, and rotational training models investigated within a harmonized methodological research programme. Despite their widespread use, few studies have directly compared these training models with respect to both functional and structural outcomes, as well as their effects on trunk mobility across different movement planes. The present manuscript represents the culmination of a multi-year prospective research programme focused on the mechanisms and applications of core stabilization training. Building on three previously published studies a structured integrative synthesis was undertaken to comparatively organize harmonized findings within a single conceptual framework (14, 15, 18). Importantly, this manuscript does not report new experimental data, systematic review procedures, or pooled re-analysis of individual-level data; rather, it provides a programme-level synthesis aimed at clarifying model-specific structural and functional adaptation patterns and translating them into a coherent conceptual framework.

Dysfunctions in core stability, such as delayed TrA activation or reduced muscle thickness observed in ultrasonographic imaging, constitute a significant clinical issue, particularly among individuals with LBP (8, 9). Although local training is frequently recommended as a method for restoring TrA function, its effectiveness beyond specific clinical indications remains controversial (19, 20). Conversely, global and rotational training approaches, which involve larger muscle groups and multiplanar movement patterns, are widely applied in both rehabilitation and motor training; however, their impact on the structural parameters of deep trunk muscles remains inconclusive (13, 21).

Therefore, the aim of the present manuscript was to develop a conceptual integrative framework describing method-specific structural and functional adaptation patterns following local, global, and rotational core stabilization training in healthy adults. A secondary aim was to discuss how these findings, derived exclusively from healthy adults, may inform future hypothesis-driven research and mechanism-oriented reasoning in rehabilitation and performance contexts.

## 2 Methods

This manuscript represents a structured integrative synthesis based on previously published prospective intervention studies conducted within a single harmonized research programme. The present manuscript does not involve new participant recruitment, new experimental procedures, systematic review methodology, or pooled re-analysis of individual-level raw data. Instead, previously reported findings and conclusions were synthesized and organized within a coherent conceptual framework describing method-specific structural and functional adaptation patterns associated with different core stabilization training approaches.

### 2.1 Data integration approach

The present manuscript provides an integrative synthesis of harmonized outcome measures derived from three previously published studies conducted within a single research programme (14, 15, 18). The original studies addressed three distinct core stabilization training concepts: local training (LT), global training (GT), and rotational training (TG) in relation to abdominal muscle thickness, trunk mobility, and functional mobility of the posterior myofascial chain. In each intervention study evaluating the effects of core stabilization training models, a non-training control group was included for comparative purposes. Only studies meeting the following criteria were included in the integrative framework:

1. healthy adult participants;
2. 4-week intervention duration;
3. identical ultrasonographic measurement protocols;
4. standardized trunk mobility assessments;
5. availability of baseline and post-intervention group mean values.

Group-level baseline and follow-up data were extracted from the original publications. Percentage changes were derived using the formula (post − baseline)/baseline × 100. Statistical significance statements correspond to those reported in the source trials. No pooled re-analysis of individual-level raw data was performed for the present manuscript.

Although the intervention protocols differed according to the investigated training model (LT, GT, or TG), all studies were conducted within the same methodological framework, including comparable intervention duration, identical ultrasonographic protocols, standardized functional assessments, and similar assessment timelines. This structured approach enables a descriptive comparison of adaptation patterns across local, global, and rotational training models while preserving methodological consistency across the analyzed interventions.

### 2.2 Characteristics of the included studies

The present conceptual framework was developed within a research programme comprising three previously published studies involving a total of 320 healthy individuals (210 women and 110 men) aged 18–45 years. Of these, 181 participants were enrolled in the core stabilization training intervention studies. The inclusion and exclusion criteria, as well as recruitment procedures, have been described in detail in the original publications (14, 15, 18) and are summarized here for completeness. Inclusion criteria comprised age between 18 and 45 years, BMI ≤ 29.9, and absence of contraindications to training. Exclusion criteria included significant spinal pain, neurological symptoms, prior abdominal surgery, chronic diseases, pregnancy, ongoing pharmacological treatment, or incomplete participation.

Ethical approval and participant consent procedures were obtained for each of the original studies, as previously reported. The overall research programme was approved by the Bioethics Committee of the Medical University of Silesia in Katowice (protocol no. KNW/0022/KB1/12/15) and registered in the ISRCTN registry (No. 82706323).

### 2.3 Measures

Outcome measures correspond to those reported in the original studies and are summarized here to facilitate comparison across intervention models.

#### 2.3.1 Lateral abdominal wall muscle thickness

- Thickness of the transversus abdominis (TrA), internal oblique (IO), and external oblique (EO) muscles was assessed using ultrasonography (USG).
- Measurements were performed using an Edan DUS 60 ultrasound system equipped with a 10 MHz linear transducer operating in B-mode.
- Ultrasound measurements followed a standardized protocol established in previous studies by the authors (14, 15, 22).
- High measurement reliability was confirmed, with ICC(3,1) values ranging from 0.98 to 0.99 (14, 15, 22).

#### 2.3.2 Trunk mobility

- Finger-to-floor test: The distance between the fingertips and the floor was measured in centimeters using a dedicated platform. Test reliability was high [ICC(3,1) = 0.99] (14, 15).
- Lateral trunk flexion test: Range of motion in the frontal plane was assessed using a Saunders digital inclinometer. The inclinometer was mounted at the midpoint of a pole held by the participant. Test reliability was ICC(3,1) = 0.97 (14, 15).

### 2.4 Design and procedures

The overall study design and assessment timeline correspond to those reported in the original intervention studies. Assessments were conducted at baseline, after 4 weeks of training, and following a detraining period, including ultrasonographic and functional evaluations. The present manuscript focuses specifically on structural and functional outcomes related to trunk stabilization within this broader research programme.

The study organization is presented in Figure 1, with assessments conducted at baseline (Measurement I), after 4 weeks of training (Measurement II), and after a 2-week detraining period (Measurement III), including ultrasonographic and functional evaluations.

**Figure 1:**
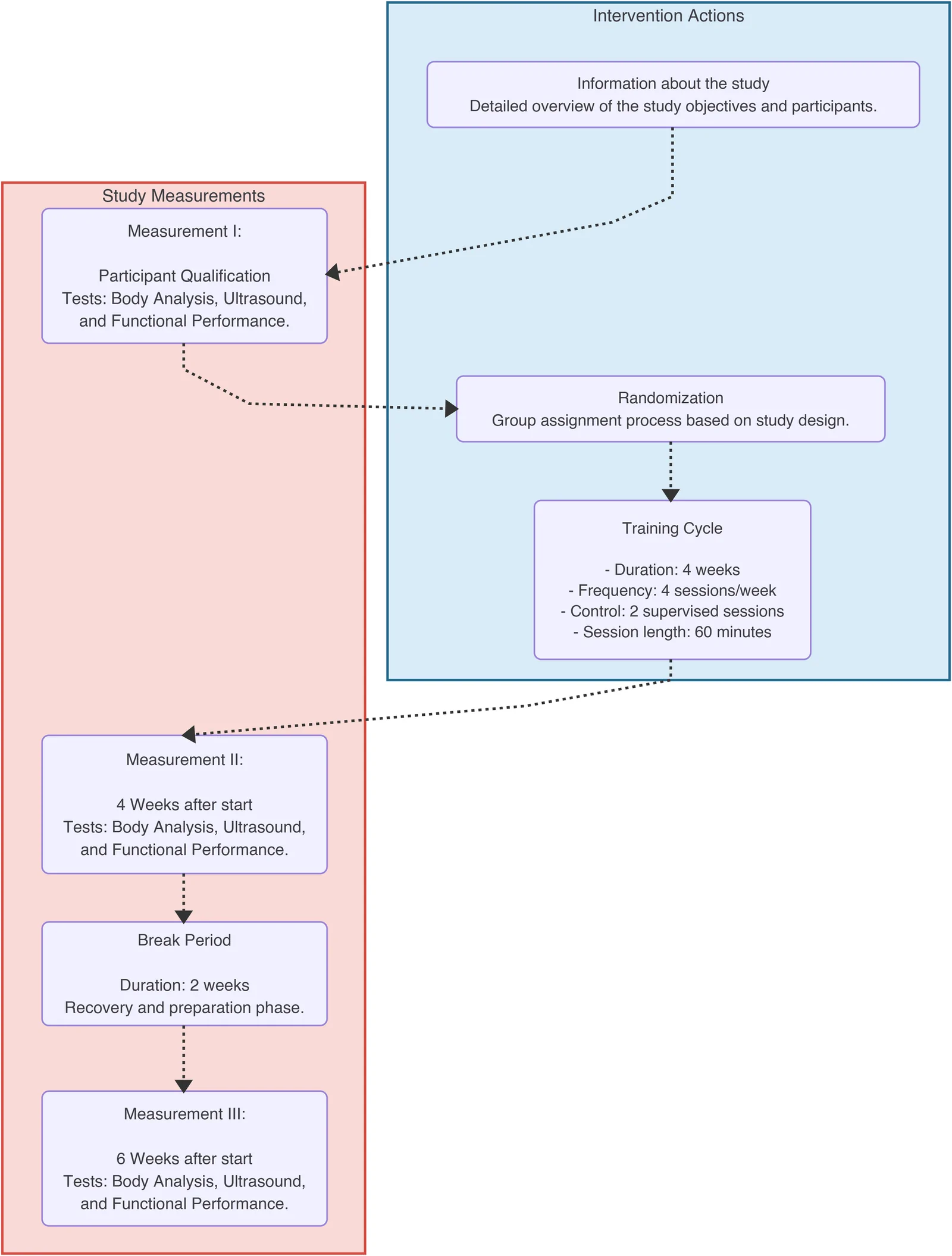
Study organization.

Figure 2 presents the overall research programme. Study 1 is included to illustrate the complete structure of the research programme on which the proposed conceptual framework is based, although it did not involve a core stabilization training intervention.

**Figure 2:**
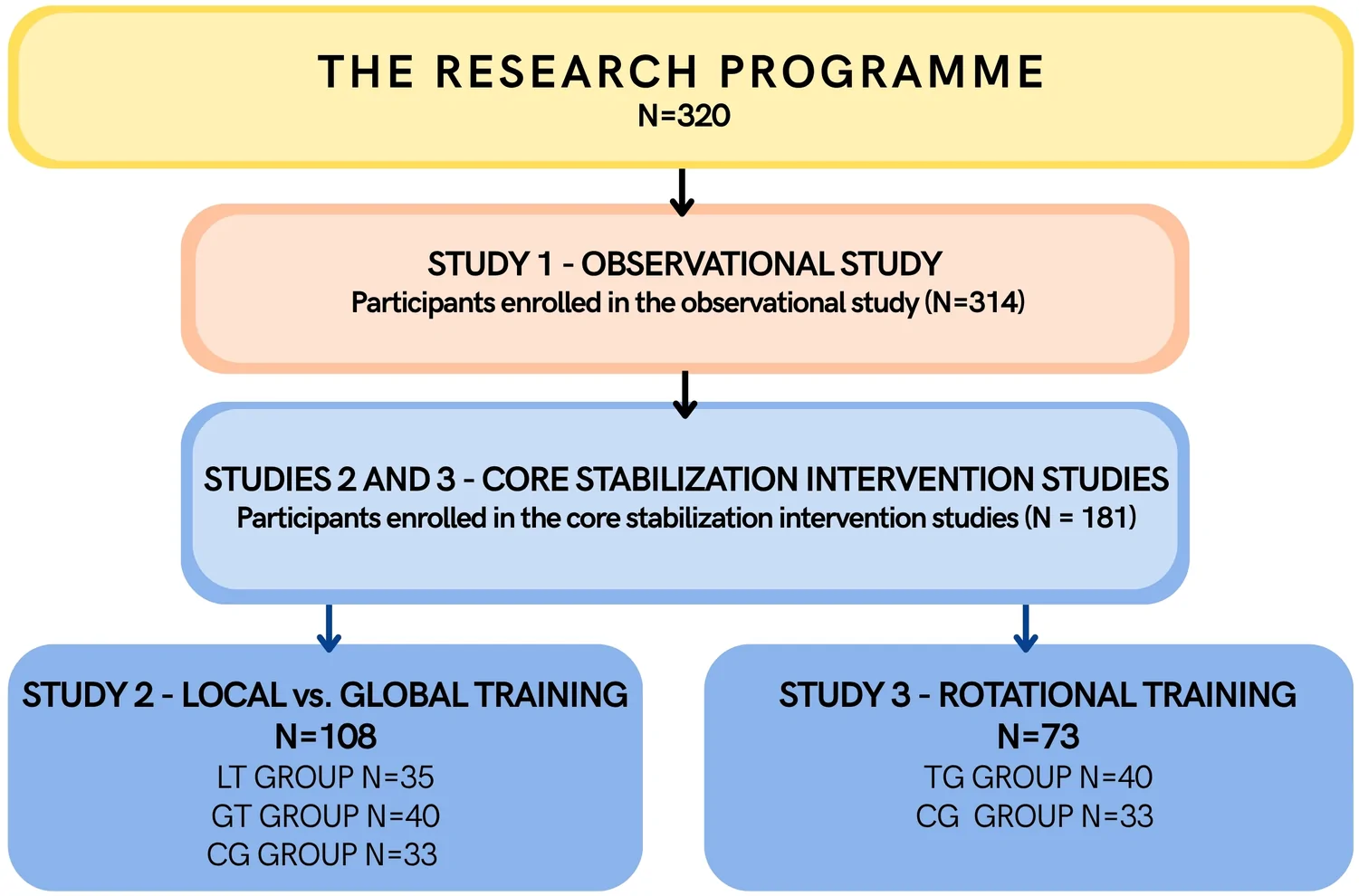
Structure of the research programme and participant distribution.

The analyses presented in the present manuscript focus specifically on structural and functional parameters related to trunk stabilization, which constitute part of a broader prospective research programme; additional outcomes have been reported elsewhere (14, 15, 18).

### 2.5 Overview of training interventions

All intervention models (LT, GT, TG) were conducted over a 4-week period and included four training sessions per week (16 sessions in total). Two sessions per week consisted of supervised group-based training conducted by the same physiotherapist in the same exercise facility, whereas two additional sessions were performed independently at home according to individualized training instructions. Participants were required to report completion of the prescribed home exercises. Each training programme was based on principles of progressive overload with continuous supervision of exercise quality, movement control, and correct exercise technique. Each session consisted of approximately 10–12 exercises performed in 1–2 sets of 10 repetitions (per side for asymmetrical exercises), with 1-minute rest between sets and 2-minute rest between exercises. All exercises were performed with respiratory control. No external resistance or additional training equipment was used during any of the interventions. All exercises were performed using body weight only, except for ultrasound imaging used as sonofeedback during selected LT exercises. The supervised sessions were conducted in groups of 8–10 participants and did not exceed 60 min.

The local training (LT) model was based primarily on isolated activation and feedforward control of the transversus abdominis muscle using palpation-guided motor control strategies and ultrasound sonofeedback (approximately 5 min per participant during each supervised training session to facilitate selective TrA activation). Participants initially learned selective activation of the TrA muscle in low positions using conscious TrA contraction without activation of other abdominal muscles during the expiratory phase. Subsequently, exercises were performed in supine and sidelying positions with progressive stages of muscle activation and breathing control, after which peripheral limb movement was gradually introduced. Exercise positions were then progressively modified from low positions through quadruped and bridging exercises to sitting, standing, and gait-related activities while maintaining anticipatory activation of the TrA muscle. Progression was achieved by gradually increasing the duration of TrA activation, progressing from low- to high-demand functional positions, and incorporating upper and lower limb movements while maintaining selective anticipatory activation of the TrA muscle.

The global training (GT) model focused on coordinated trunk muscle activity without isolated contraction of deep stabilizing muscles. The programme incorporated functional movement patterns including various forms of prone, side, and supine bridging exercises, stabilization tasks in quadruped positions, and squat exercises. Exercises were performed with breathing control, supervision of movement pattern quality, and gradual progression of movement complexity. Progression was achieved by transitioning from easier to more demanding exercise modifications, reducing the base of support, increasing lever length, and gradually increasing the number of exercise sets according to the participants' performance while maintaining correct movement quality.

The rotational training (TG) model focused primarily on multiplanar trunk movement and lumbar spine rotational control performed both in supine lying and quadruped positions. The programme included global rolling patterns initiated by upper or lower limb movement as well as “monkey-type” exercises. The intervention was based on gradual progression of rotational movement complexity while maintaining correct movement technique and breathing patterns. The intervention was based on gradual progression of rotational movement complexity by reducing the base of support, increasing lever length and movement speed, and progressing from simpler to more demanding multiplanar movement patterns while maintaining correct movement technique and coordinated breathing patterns.

Detailed descriptions of the training protocols, including representative illustrations of the exercise positions, are available in our previous publications (14, 15).

### 2.6 Statistical analysis

Statistical analyses correspond to those reported in the original studies. The present manuscript does not involve new statistical modelling or pooled analysis of individual-level data. Instead, reported outcomes are interpreted comparatively across intervention models to identify consistent patterns of structural and functional adaptation.

## 3 Synthesized findings

The following section provides an integrative evidence synthesis based on previously published intervention studies included in the present conceptual framework. By descriptively examining harmonized structural and functional outcomes across training models, the synthesis aims to identify consistent adaptation patterns and their potential implications for rehabilitation and performance contexts. Statistical interpretations correspond to those reported in the original studies, and no pooled analysis of individual-level data was performed.

### 3.1 Lateral abdominal wall muscle thickness

The analysis revealed training-specific changes in lateral abdominal wall muscle thickness (Figure 3), reflecting distinct patterns of muscular adaptation across the applied interventions.

**Figure 3:**
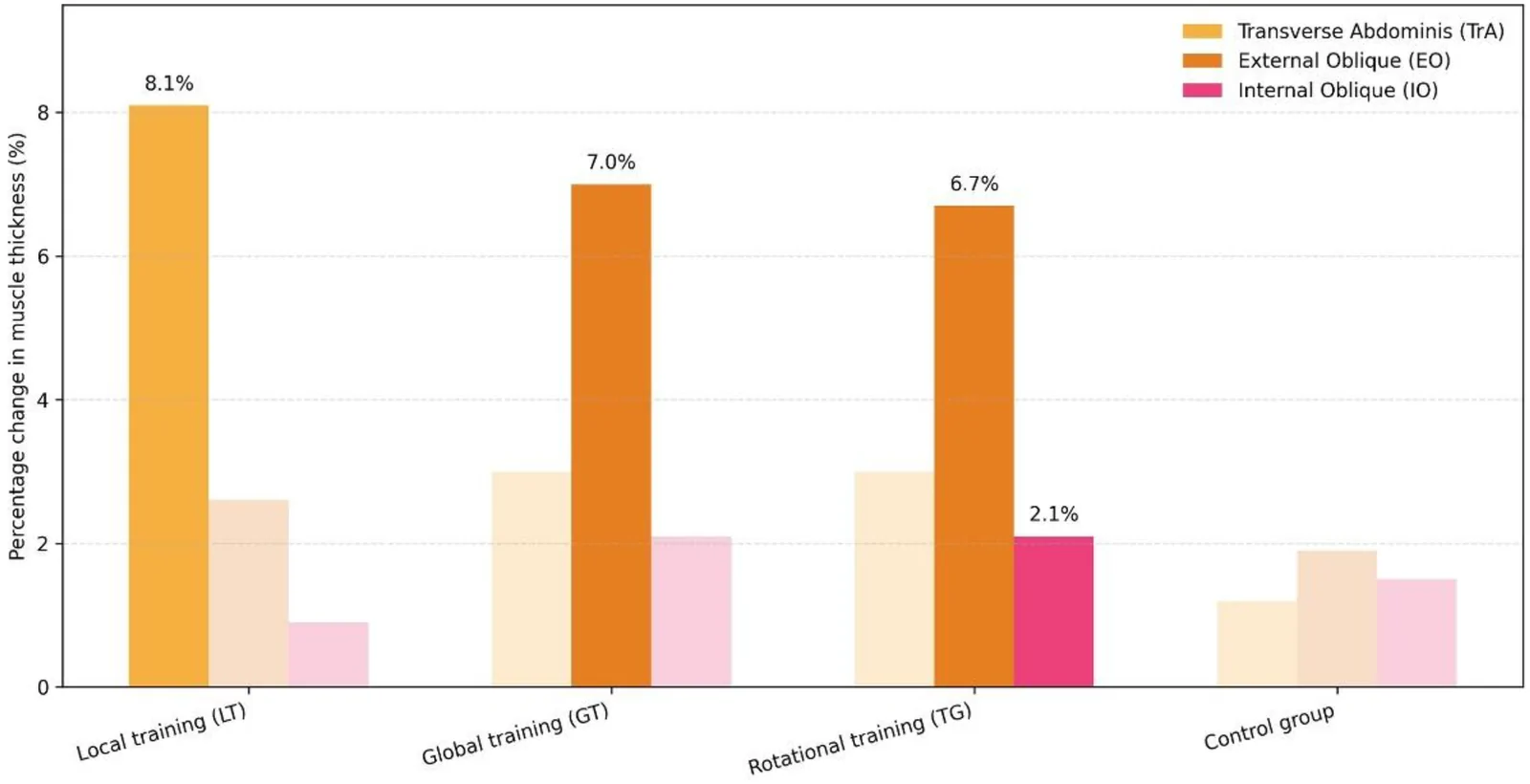
Training-specific changes in lateral abdominal wall muscle thickness across intervention models (14, 15).

In the local training (LT) group, a statistically significant increase in transversus abdominis (TrA) thickness was observed between Measurements I and III (*p* = 0.0015), while no significant changes were detected in the remaining lateral abdominal wall muscles (14).

In the global training (GT) group, a significant increase in external oblique (EO) muscle thickness was identified (*p* < 0.0001). In addition, a statistically significant increase in the combined TrA + IO + EO muscle complex was observed (*p* = 0.0026), indicating a global adaptation of the lateral abdominal wall musculature (14).

In the rotational training (TG) group, statistically significant increases were found in the thickness of the internal oblique (IO) and external oblique (EO) muscles (*p* < 0.01), whereas no significant change was observed in TrA thickness (15).

In the control group, no statistically significant changes in lateral abdominal wall muscle thickness were observed (14, 15).

Changes in TrA, IO, and EO muscle thickness across training groups are illustrated in Figure 3, illustrating distinct adaptation patterns observed following LT, GT, and TG interventions.

Percent values represent within-group changes between baseline and follow-up (Measurement I–III), calculated from group mean values reported in the original studies. Visually deemphasized bars indicate changes that were not statistically significant.

### 3.2 Trunk mobility

Trunk mobility changes were evaluated in two movement planes and are presented in Figure 4:

**Figure 4:**
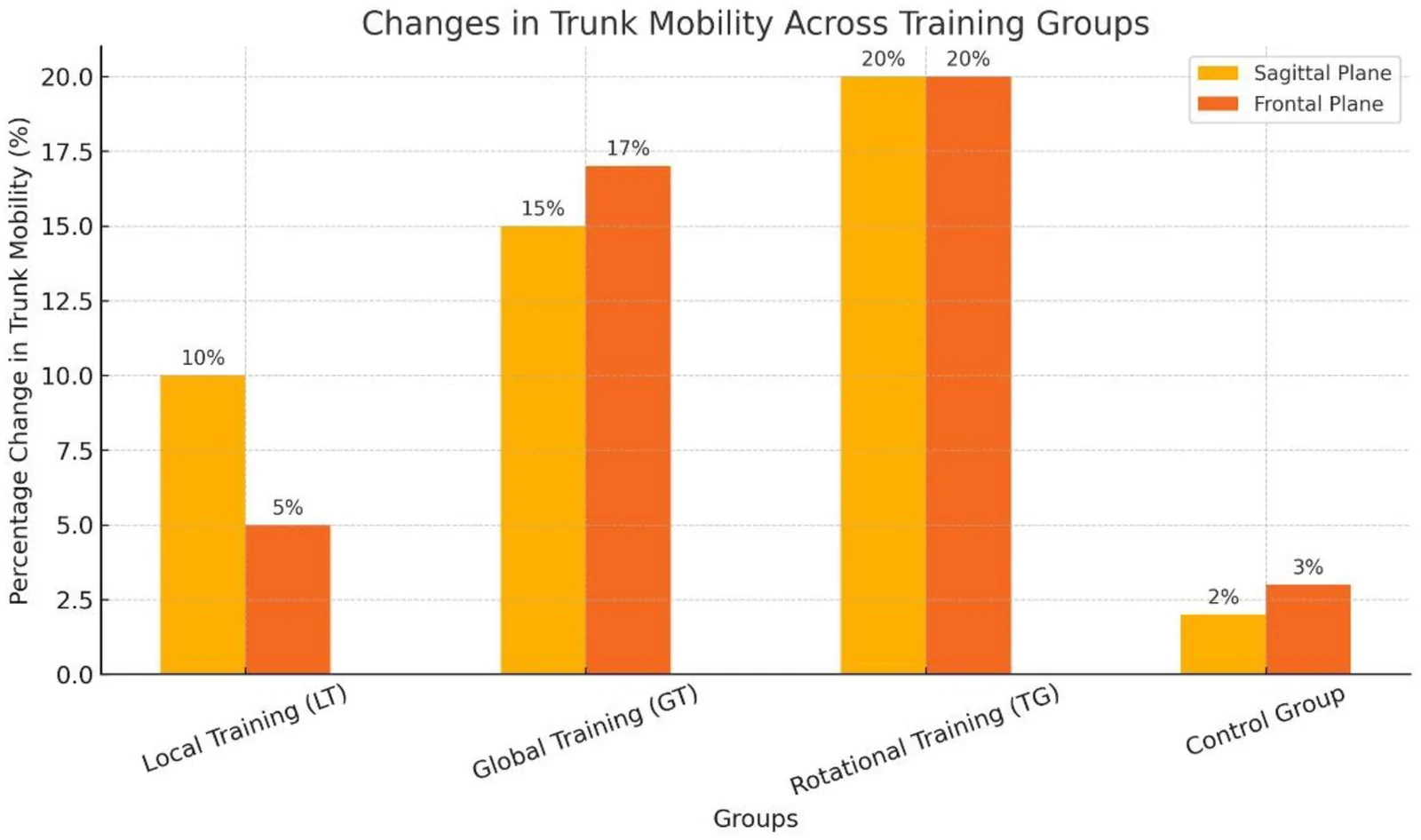
Training-specific changes in trunk mobility across intervention models (14, 15).

Sagittal plane: The finger-to-floor test demonstrated improved trunk mobility in all intervention groups. Statistically significant improvements were observed in the LT, GT, and TG groups. (*p* < 0.01). A small but statistically significant improvement was also observed in the control group (*p* < 0.05), likely reflecting a test–retest effect (14, 15).

Frontal plane: The lateral trunk flexion test demonstrated statistically significant improvements in trunk mobility in the LT, GT, and TG groups (*p* < 0.01). Smaller but statistically significant improvements were also identified in the control group (14, 15).

Small but statistically significant improvements observed in the control group likely reflect a learning or test–retest effect rather than a training-related adaptation.

### 3.3 Follow-up analysis

Repeated measurements performed 2 weeks after completion of the training program (difference between Measurements II and III) demonstrated maintenance of positive effects in the LT, GT, and TG groups. The findings indicate stabilization of muscle trophic adaptations and sustained improvements in trunk mobility.

## 4 Discussion

Direct comparisons between local, global, and rotational core stabilization models are still limited in the available literature, especially in studies using the same ultrasonographic and functional assessment methods.

### 4.1 Changes in lateral abdominal wall muscle thickness

The results of the study indicate that each core stabilization training modality influences changes in lateral abdominal wall muscle thickness, with the underlying mechanisms differing depending on the applied training model (Figure 3).

In the LT group, a significant increase in transversus abdominis (TrA) thickness was observed. This finding is consistent with previous studies emphasizing the effectiveness of isolated local training in activating the TrA, particularly when supported by sonofeedback-based interventions (5, 14). The neurophysiological mechanism underlying this adaptation is associated with improved motor unit recruitment based on feedforward control, which has been reported to play a crucial role in the rehabilitation of patients with impaired core stability, such as those with chronic low back pain (LBP).

In contrast, the GT group demonstrated increased thickness of the external oblique (EO) muscle, accompanied by a significant increase in the combined TrA + IO + EO muscle complex reported in the original study. These findings are in line with the observations of Park and Yu (20), who suggested that global stabilization exercises promote hypertrophy of superficial trunk muscles while exerting limited influence on TrA thickness. The increased hypertrophic response of global muscles is likely attributable to the involvement of larger muscle groups during dynamic movement patterns, which may contribute to overall functional trunk stability.

Rotational training (TG) produced distinct effects, characterized by increased thickness of the oblique abdominal muscles (IO and EO). Dynamic movements performed in the transverse plane preferentially engage the oblique muscles, supporting previous evidence regarding their role in controlling rotational movements and contributing to dynamic trunk stability (13). However, the absence of significant TrA adaptations in the TG group suggests that isolated activation of this muscle is not a primary objective of rotational training and should not be interpreted as such.

Nevertheless, interpretation of structural adaptations within core stabilization research remains complex. Although transversus abdominis activation is widely considered an important component of trunk stabilization and feedforward motor control mechanisms (4–6), recent reviews have emphasized that changes in lateral abdominal muscle thickness and activation may be influenced by methodological factors related to exercise prescription, including intervention duration, training frequency, and exercise volume (23). Both previous literature and the present findings suggest that structural adaptations should not be interpreted as isolated indicators of functional improvement or clinical effectiveness and may require complementary functional assessment for appropriate interpretation (24, 25). Moreover, current evidence does not support the universal superiority of any single stabilization strategy, suggesting that adaptation patterns may depend on the specific training objective, movement demands, and individualized functional assessment of trunk stabilization deficits and motor control characteristics (23, 25).

### 4.2 Trunk mobility

All training groups demonstrated improvements in trunk mobility, consistent with previous findings on core stabilization interventions (26). Pronounced improvements were observed following GT and TG interventions, indicating that dynamic, multiplanar exercises may contribute to enhanced trunk mobility. A slight improvement in trunk mobility was also observed in the control group, likely reflecting a familiarization effect related to repeated testing; however, this change was minimal and did not influence the overall study outcomes. Nevertheless, repeated functional testing itself may induce a learning effect that should be taken into account when interpreting intervention effects in future studies (15).

Rotational training (TG) resulted in a significant improvement in frontal-plane mobility, suggesting its potential application in the rehabilitation of individuals with limitations in lateral and transverse movements. These findings support previous reports indicating that rotational movements activate the oblique and paraspinal muscles, which may be of particular relevance in clinical populations presenting with unilateral movement restrictions (7, 27).

### 4.3 Implications for future research

The applied assessment methods including ultrasonography and trunk mobility tests demonstrated high reliability and may be useful for future research and standardized assessment protocols (14, 15).

### 4.4 Study limitations

Several limitations of the present study should be acknowledged. Half of the training sessions were performed independently by the participants. Despite prior instruction, supervision, and remote monitoring, this may have influenced exercise technique and execution quality. In addition, the study population consisted exclusively of healthy individuals, which limits the generalizability of the findings to other populations, such as patients with chronic low back pain. Furthermore, as the present manuscript integrates data derived from intervention studies conducted within a single research programme, external validation across independent cohorts is warranted.

### 4.5 Conceptual stage-based framework based on integrated evidence

Current concepts of core stabilization describe trunk rehabilitation as a progressive process involving motor control, muscular coordination, and increasingly functional movement patterns. Previous theoretical models have emphasized the complementary roles of local stabilization, global force transmission, and task-specific movement integration. However, these concepts have rarely been interpreted together with objective evidence describing muscle-specific structural adaptations.

Within our research programme, local, global, and rotational core stabilization exercises demonstrated distinct structural and functional adaptation patterns in healthy adults. Local stabilization predominantly increased transversus abdominis thickness, global stabilization preferentially enhanced the external oblique and overall lateral abdominal wall musculature, whereas rotational training was associated with adaptations of the oblique muscles accompanied by improvements in multiplanar trunk mobility. These findings should be interpreted as training-specific adaptations rather than evidence supporting the superiority of one exercise strategy over another.

Integrating these observations with existing theoretical concepts led us to propose the stage-based framework presented in Figure 5. This framework represents a conceptual hypothesis suggesting that different structural and functional dysfunction profiles may ultimately require different rehabilitation priorities. Although this hypothesis cannot be verified based on the present studies in healthy adults, it provides a biologically plausible rationale for future prospective research examining whether specific dysfunction patterns are associated with differential responses to targeted core stabilization strategies in clinical populations. Accordingly, Figure 5 should be interpreted as a hypothesis-generating conceptual framework rather than a validated clinical model or rehabilitation guideline.

**Figure 5:**
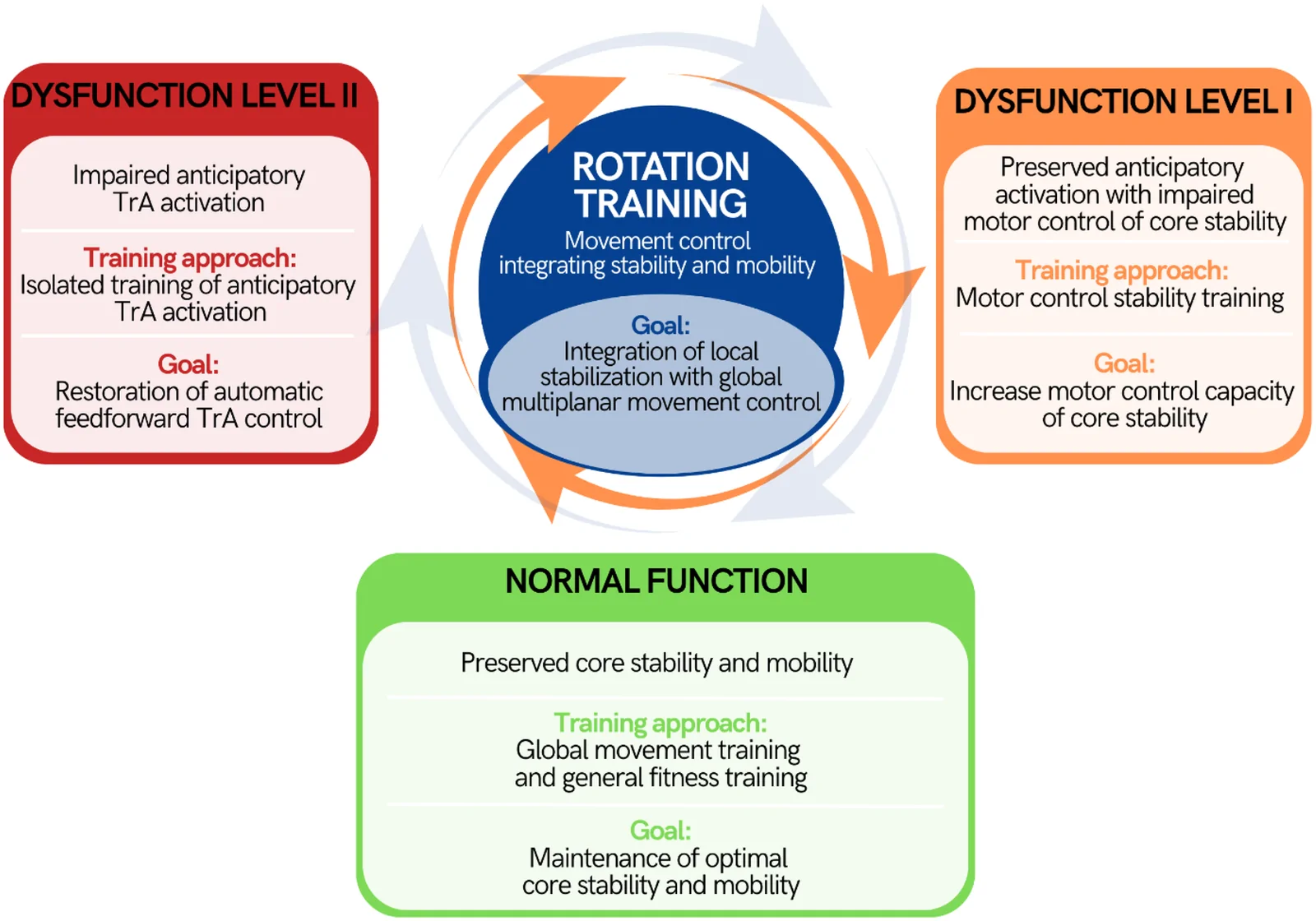
Proposed hypothesis-generating conceptual framework for stage-based core stabilization strategies.

### 4.6 Hypothesized selection of training strategies based on dysfunction profiles

The following training considerations are derived from the structural–functional adaptation patterns observed within the present research programme and are interpreted in the context of extensively documented research on trunk motor control and stabilization mechanisms that underpin contemporary training concepts. They represent a structured synthesis of established scientific knowledge contextualized by the present findings and are intended to provide a mechanism-informed rationale for training selection rather than formal clinical guidelines.

The selection of an appropriate training method should be guided by comprehensive functional assessment and identification of individual motor deficits.

#### 4.6.1 Local training (LT)

- Observed Adaptations: Associated with increased transversus abdominis thickness in healthy adults.
- Hypothesized Dysfunction Profile: May represent a hypothesized dysfunction profile characterized by impaired anticipatory TrA activation or reduced deep trunk control.
- Potential Injury Prevention and Performance Contexts: The observed adaptations may be relevant to activities requiring precise anticipatory trunk stabilization, segmental motor control, and efficient lumbopelvic coordination.

#### 4.6.2 Global training (GT)

- Observed Adaptations: Associated with increased thickness of the external oblique abdominal muscle and the combined TrA + IO + EO muscle complex, accompanied by improved trunk mobility in the sagittal and frontal planes in healthy adults.
- Hypothesized Dysfunction Profile: May represent a hypothesized dysfunction profile characterized by reduced overall trunk stability, impaired trunk force transmission, and limited functional movement integration.
- Potential Injury Prevention and Performance Contexts: The observed adaptations may be relevant to activities and sports requiring dynamic, multidirectional movements, efficient force transmission, and whole-body movement integration.

#### 4.6.3 Rotational training (TG)

- Observed Adaptations: Associated with improved trunk mobility in the frontal and sagittal plane and increased thickness of the oblique abdominal muscles (IO and EO) in healthy adults.
- Hypothesized Dysfunction Profile: May represent a hypothesized dysfunction profile characterized by restricted trunk mobility and impaired rotational movement control, potentially affecting gait patterns.
- Potential Injury Prevention and Performance Contexts: The observed adaptations may be particularly relevant to activities and sports involving rotational movements, rapid changes of direction, and multiplanar trunk control. Owing to its combined effects on oblique muscle adaptation (including the internal oblique) and trunk mobility, rotational training may also conceptually complement both local and global stabilization strategies.

### 4.7 Integration of training modalities

Selection of the training modality should therefore be preceded by individualized functional assessment aimed at identifying deficits in deep trunk muscle activation, trunk muscle weakness, and disturbances in lumbopelvic motor control.

A staged progression beginning with local training when deep trunk activation is a primary objective, followed by incorporation of global and rotational components, may represent a conceptual staged approach consistent with principles of load progression and motor integration.

Notably, the combined structural adaptations involving the internal and external oblique muscles together with improvements in trunk mobility suggest that rotational training may conceptually bridge local and global stabilization strategies. This unique adaptation profile raises the hypothesis that rotational training could serve as a transitional component within staged rehabilitation programmes, complementing both local and global stabilization strategies.

#### 4.7.1 Long-term effects of training

The obtained results indicate stabilization of training effects following a 4-week intervention program in healthy adults. Within the study period, improvements in muscle morphology and trunk mobility remained stable following the 4-week intervention in healthy adults.

#### 4.7.2 Summary of practice implications

The study findings provide a structured framework for selecting and sequencing core stabilization training modalities. The applied assessment methods—including ultrasonography and trunk mobility tests—demonstrated high reliability and may provide useful tools for both clinical assessment and future research (14, 15). Future studies should evaluate the translational effects of these training models in specific clinical populations and explore advanced imaging techniques, such as MRI, for objective analysis of structural tissue adaptations.

While the present framework provides a structured synthesis of harmonized evidence, further research is needed to determine its applicability in clinical populations and to evaluate long-term adaptation patterns. Future investigations integrating functional assessment with advanced imaging modalities may further clarify the mechanisms underlying core stabilization training and support individualized rehabilitation strategies.

## 5 Conclusions

This study provides an integrated framework for understanding method-specific structural and functional adaptations resulting from different core stabilization training approaches in healthy adults. The findings suggest that no single training model appears universally superior, as local, global, and rotational approaches demonstrated distinct structural and functional adaptation profiles. By combining ultrasonographic assessment with functional outcomes, the proposed framework may support mechanism-informed individualized training selection based on targeted adaptation profiles rather than universal application of a single stabilization strategy.

The presented synthesis contributes to mechanism-informed decision-making in rehabilitation and performance contexts; however, its direct applicability to clinical populations requires further empirical validation.

## Data Availability

The original contributions presented in the study are included in the article and the original source articles. Further inquiries can be directed to the corresponding author.
